# Regular Yoga Modulates Attention Bias During the Luteal Phase in Women with Premenstrual Syndrome

**DOI:** 10.3390/brainsci16010036

**Published:** 2025-12-26

**Authors:** Xue Li, Danyang Li, Ying Liu, Chenglin Zhou, Xiaochun Wang

**Affiliations:** 1 School of Psychology, Shanghai University of Sport, Shanghai 200438, China; lixue@sus.edu.cn (X.L.); liuying@sus.edu.cn (Y.L.); zhouchenglin@sus.edu.cn (C.Z.); 2 School of Physical Education, Nanjing Xiao Zhuang University, Nanjing 211171, China; susldy0918@hotmail.com

**Keywords:** regular yoga, attention bias, premenstrual syndrome

## Abstract

**Objectives**: Women with Premenstrual Syndrome (PMS) tend to exhibit an excessive attention bias toward negative stimuli during the luteal phase. This study intends to investigate the effect of regular yoga on attention bias of women with PMS during the luteal phase and explore the mechanisms underlying such changes. **Methods:** Sixty-four women with PMS were recruited, coded and randomly assigned to either a 12-week yoga group (n = 32) or a control group (n = 32). The dot-probe task was used to assess attention bias at baseline and 12 weeks later. Data analysis was performed using SPSS 27.0 software, with analytical methods including descriptive statistics, repeated-measures analysis of variance (RM-ANOVA), simple effect analysis, cluster-based permutation test and Pearson correlation analysis. The Holm–Bonferroni method was used to correct for multiple comparison errors. **Results:** RM-ANOVA revealed significant time × group interaction effects for attention orientation, attention disengagement, P1 component, and P3 component. Simple effect analysis indicated that, compared with the control group, the yoga group exhibited significant modulations in attention orientation (t = −7.33, *p* < 0.001), P1 (t = 8.94, *p* < 0.001), attention disengagement (t = 6.89, *p* < 0.001), and P3 (t = 4.42, *p* = 0.002) after 12 weeks of intervention. Cluster-based permutation tests demonstrated that the yoga group showed significant reductions in P1 and P3 amplitudes after 12 weeks. Pearson correlation analysis indicated that attention orientation was significantly negatively correlated with P1 amplitude, while attention disengagement was significantly positively correlated with P3 amplitude. **Conclusion:** Regular yoga can regulate the behavioral indicators and electroencephalographic (EEG) indicators related to attention bias and exerts a positive effect on modulating attention bias toward negative stimuli in women with PMS during the luteal phase.

## 1. Introduction

A woman’s menstrual cycle typically includes four phases: the luteal phase, menstrual phase, follicular phase, and ovulatory phase [1,2]. In 1931, Frank named the specific syndrome characterized by negative mood changes during the luteal phase as Premenstrual Syndrome (PMS) [3]. Women with PMS exhibit persistent and severe negative mood changes during the luteal phase, including depressive, anxious, irritable, and other negative emotional symptoms, accompanied by physical symptoms such as breast pain, abdominal pain, headache, and limb swelling. These emotional and physical symptoms resolve spontaneously during the menstrual phase [4].

Studies indicate that negative mood changes in women with PMS during the luteal phase are significantly influenced by attention bias [5,6,7,8,9]. Regarding the consistency of findings, multiple studies converge on a key observation: women with PMS exhibit a pronounced attention bias toward negative stimuli specifically during the luteal phase, with no such bias detected in the menstrual or follicular phases [10]. Women with PMS show an excessive attention bias toward negative emotions during the luteal phase, being particularly sensitive to negative stimuli in the environment and inclined to focus on them. Women with PMS exhibited a greater attention bias for negative facial expressions during the luteal phase compared to healthy women [7]. Negative emotions increased emotional interference and women with PMS showed a stronger attention bias toward negative emotional stimuli [8]. Women with PMS exhibited higher levels of self-focus in negative emotional states compared to healthy individuals [9]. In terms of effect magnitude, while direct quantitative comparisons are rarely explicitly reported across studies, the consistency of directional findings across different methodologies (e.g., facial expression paradigms, self-focus assessments, emotional interference tasks) implies a moderate-to-robust effect of the luteal phase on attention bias in PMS. Notably, Baker et al. [11] provided neurophysiological support for this phenomenon: their electroencephalographic (EEG) data revealed increased beta (β1) frequency and P3 amplitude in women with PMS across two menstrual cycle phases, alongside self-reported drowsiness, fatigue, and psychomotor slowing. However, several critical limitations undermine the current evidence base: most studies rely on small-to-moderate sample sizes, limiting the generalizability of findings and precluding rigorous assessments of whether attention bias varies by PMS severity; methodological heterogeneity—including differences in attention bias tasks and phase-timing protocols—makes direct comparisons of effect magnitudes across studies challenging.

Various paradigms have been used to explore attention bias, including the dot-probe task, eStroop task, emotional spatial cueing paradigm, and visual search task, which assess different aspects of attention [12]. The dot-probe task measures the extent to which threatening stimuli attract attention [13,14]. Although the behavioral performances of these tasks may differ, there are some commonalities at the neural level. For example, early, middle, and late event-related potentials (ERPs) can be elicited by both threatening stimuli and stimuli presented in emotional contexts. Bar-Haim et al. conducted an extensive meta-analysis of 172 behavioral attention bias studies and found that only individuals with depression and anxiety exhibited significant threat-related biases in paradigms such as the eStroop task, dot-probe test, and emotional spatial cueing, whereas healthy individuals did not show such biases, and the degree of bias was similar across different anxiety populations. Most ERP components related to attention bias are influenced by emotional stimuli and spatial attention [15]. Early components capture the initial stages of attention processing and are suitable for exploring more automatic forms of attention bias; late components capture the later stages of attention processing and may thus reflect more strategic forms of attention bias. P1, typically the first major visual ERP component, is thought to originate from the extrastriate visual cortex, with maximal amplitude at lateral occipital electrode sites. It usually begins 60–90 ms after stimulus presentation and peaks between 100 and 130 ms [16]. Allocation of attention to a stimulus leads to an increase in P1 amplitude [17]. P3, first identified by Sutton et al. with a peak at approximately 300 ms, is also known as the late positive potentialand is considered a neural index of sustained attention to emotional stimuli [18,19,20]. P3 amplitude is influenced by the amount of attentional effort allocated to a stimulus, with larger amplitudes observed when participants devote more attentional effort.

Yoga is a Sanskrit word, meaning the connection of the mind, body, emotions, logic, and attention to the action. Yoga exercise is composed of physical (asana), breathing (pranayama), and mental (pratyahara) practices, resulting in health, relaxation, and positive awareness [21]. Physiologically, yoga relaxes the nervous system, balances the endocrine system, increases blood and oxygen flow to reproductive organs, and purifies the liver and surrounding muscles; psychologically, it reduces stress and enhances the hypothalamus’ ability to regulate hormones. Yoga intervention studies on PMS have been conducted in Asian countries, including India, Iran, Turkey, etc. Studies have shown that regular practice of basic yoga sequences or energy activation sequences could reduce the frequency of PMS; compared with the pre-intervention state, yoga helps relieve pain symptoms [22,23,24,25], reduce stress [22,26], and has a positive impact on sleep disorders [27], memory loss [28] and depression [29]. Studies have shown that yoga might help to alleviate emotional problems in women with PMS; however, the underlying neural mechanisms of the improvement remain unclear [23,24,30,31]. A synthesis of these 10 relevant studies reveals notable variability in yoga intervention lengths: 4 studies had a sample size of fewer than 30 participants, another 4 had a sample size of over 30, and 2 had a sample size of over 50. The age of the participants ranged from 16 to 45 years old, and 90% of the studies adopted a yoga practice frequency of 2–3 times per week. 1 study each adopted 4-week, 6-week, and 8-week protocols, 2 studies used a 10-week design, and the largest subset (5 studies) implemented a 12-week intervention. While even short-term practice shows benefits—for instance, 4 weeks of regular yoga has been demonstrated to reduce PMS symptoms in affected women—these effects are often modest and lack long-term sustainability.

Based on the preceding analysis, this study intends to investigate how regular yoga modulates attention bias during the luteal phase in women with PMS. To strengthen the potential of our intervention to target the underlying mechanisms of PMS-related attention bias (e.g., by improving emotional regulation and neural processing of negative stimuli), and to build on the most consistent and impactful findings from prior research, the present study selected a 12-week yoga intervention protocol. We hypothesize that: (1) 12-week regular yoga practice will ameliorate excessive negative attention bias in women with PMS during the luteal phase, as reflected by reduced attention orientation toward negative stimuli and enhanced attention orientation from negative stimuli; (2) these behavioral improvements will be accompanied by normalized neural activity, characterized by decreased P1 amplitude (attenuated early sensory vigilance to negativity) and adjusted P3 amplitude (enhanced late cognitive attentional control). To verify these hypotheses, we employed a randomized controlled design with a yoga intervention group and a waitlist control group, combining the dot-probe task (behavioral attention bias) and EEG/ERP recordings (neural correlates) at baseline and post-intervention. Our core findings confirm that yoga exerts a dual regulatory effect on both behavioral attention bias and its underlying neural mechanisms, highlighting the potential of yoga as a non-pharmacological intervention for PMS by targeting the attentional control pathway.

## 2. Materials and Methods

### 2.1. Research Design

The study was conducted at Shanghai University of Sport from December 2023 to December 2024 and approved by the Ethics Committee of Shanghai University of Sport on 23 December 2022. The EEG experiments were performed in the Experimental Center of the School of Psychology, while the yoga program was conducted in the comprehensive training room of the Leisure Pavilion. Following the principle of voluntary participation, participants signed informed consent forms for full participation, including the EEG experiments.

The exercise group (yoga intervention group) participated in a 12-week yoga practice, while the control group maintained their usual exercise habits. The exercise status of participants in both groups was strictly controlled and monitored. Exercise Group: To ensure the compliance with the intervention and the standardization of exercise intensity, the study adopted the following monitoring methods: recording the attendance of each participant and the duration of each exercise session; if a participant was absent once, she was required to make up for the missed session through an online video course within 48 h to ensure that the total exercise volume met the standard; coaches and teaching assistants conducted follow-ups with participants after each exercise session to ensure that the exercise was carried out at a moderate intensity. Control Group: Participants were required to maintain their usual exercise habits before enrollment; through 1–2 follow-ups per month, the study controlled that participants did not participate in any form of structured yoga practice or other new exercise programs that might affect PMS symptoms during the intervention period.

**Figure 1:**
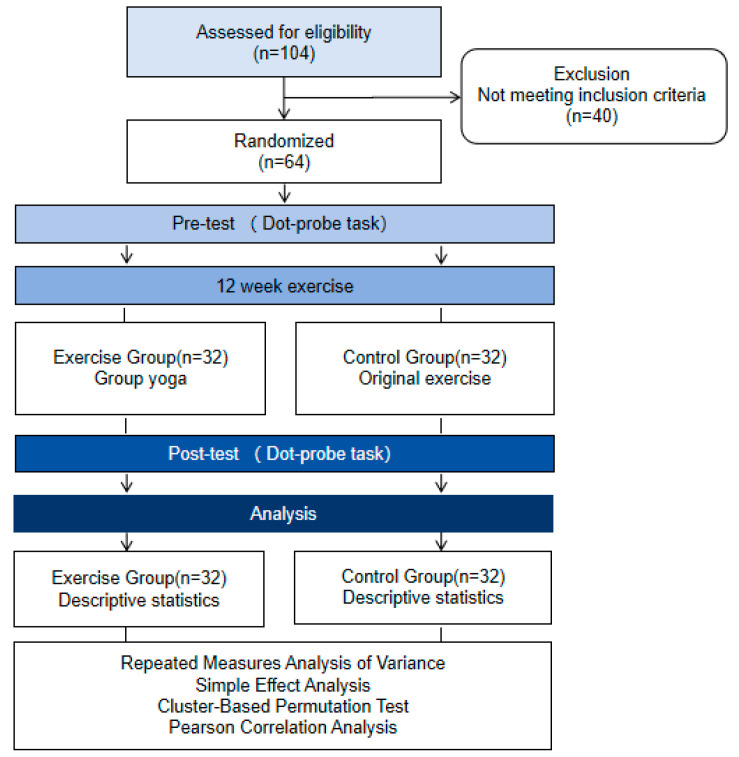
Research procedure.

Both groups completed pre-test and post-test assessments at baseline and 12 weeks later. Participants received monetary compensation after the experiment. The research procedure is shown in Figure 1.

### 2.2. Participants

Female college students with PMS were recruited using the Premenstrual Symptoms Screening Tool (PSST) and Physical Activity Rating Scale (PARS), with the following criteria: regular menstrual cycles, no significant intergroup differences in exercise intensity, duration and frequency, and exclusion of those with premenstrual dysphoric disorder (PMDD)—the severe form of PMS. The PSST consists of 19 items, each scored on a 4-point scale, and can distinguish between PMS and PMDD based on statistical criteria. Studies have demonstrated that the PSST has a reliability of 0.91 [32], while the PARS has a reliability of 0.80 [33]. A total of 104 female college students with PMS were recruited as the initial sample. Inclusion criteria: (1) Aged 18–32 years; (2) Normal body mass index (BMI); (3) General health status without immune system or reproductive system diseases; (4) Regular menstrual cycles with no history of hormonal medication use; (5) No regular yoga practice (defined as less than once per month in the past 6 months); (6) Voluntary participation and willingness to complete the trial. Exclusion criteria: (1) Lack of informed consent or unwillingness to participate; (2) Development of any medical condition or circumstance that would preclude continued yoga practice (resulting in withdrawal from the study). 

After enrollment, each participant was randomly assigned a unique numerical code and then divided into the exercise group and the control group via random drawing, with 32 participants in each group. A single-blind design was adopted in this study. Blinding was implemented as follows: the researcher responsible for envelope preparation was not involved in participant recruitment or data collection; the primary researcher in charge of enrollment and intervention assignment, data analyzer, and participants were unaware of the envelope contents and group allocation. Throughout all stages, assessors only had access to numerical codes and could not obtain associated information such as participants’ names or intervention protocols. All participants could contact researchers by telephone for consultation at any time, and researchers conducted two telephone follow-ups to ensure completion of assessment questionnaires.

Sample size was calculated based on the methods described by [34]. The study anticipated that the yoga intervention would yield a medium-to-large effect size. Based on this expectation, and using G*Power 3.1 with a significance level of 5% and 80% statistical power, it was calculated that a minimum of 22 participants per group would be required. Ultimately, each group retained 32 valid participants, which aligns with the projected effect size. There were no significant differences between the exercise group and the control group in terms of height, age, weight, and BMI; PMS-related psychological dimension, physical dimension, impact dimension and all symptoms and impacts (assessed by PSST); exercise intensity, exercise time and exercise duration (assessed by PARS). The baseline characteristics of participants are shown in Table 1.

### 2.3. Yoga Intervention

**Figure 2:**
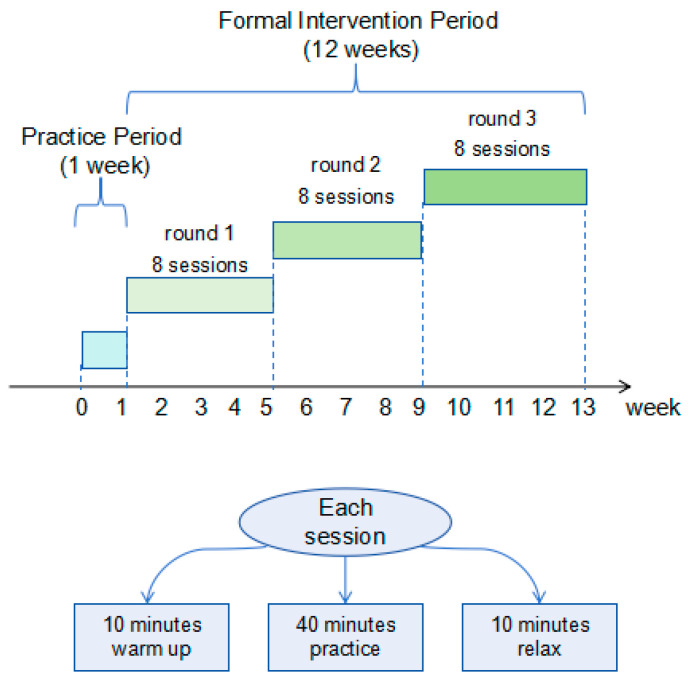
Yoga programme.

A 12-week moderate-intensity group yoga program with 2 sessions per week was designed and implemented. The yoga program was jointly designed by two experienced yoga instructors on the research team, based on the symptoms of PSST and participants’ verbal reports of the locations and prevalence of premenstrual physical pain. This program integrated content targeting physical pain relief with breathing exercises and meditation practices to promote physical and mental relaxation, while ensuring that the exercise intensity was maintained at a moderate level (see Figure 2). The yoga program was structured around 8 distinct themes and delivered in a cycle of 3 rounds. The difficulty of the yoga gradually increased as participants mastered the skills. Each session included: 10 min of meditation and warm-up, 40 min of breathing and asana practice, and 10 min of relaxation and deep rest. Yoga practices are taught by two senior yoga instructors. Before the yoga practice, conduct a one-week warm-up practice to help the instructor understand the condition of the subjects. During the yoga practice, the instructor demonstrates the skill requirements and techniques for each subject, and the trainees follow and practice. The instructor provides personalized guidance based on the subjects’ conditions, offering alternative poses and auxiliary protective equipment to meet the needs of subjects with different flexibility levels.

### 2.4. Experimental Task

In this study, the dot-probe task was used to explore the behavioral characteristics and EEG characteristics of participants’ attention bias. All experiments were conducted during the luteal phase of the participants. The luteal phase was determined based on participants’ self-reported menstrual cycle records, specifically defined as the week preceding the next menstrual period. A follow-up survey on participants’ actual menstruation time was conducted after the experiment to confirm that all experimental procedures were completed during the luteal phase.

All experiments were conducted in the same laboratory at the Experimental Center of the School of Psychology, using identical computer equipment. The temperature, humidity, and lighting conditions were strictly standardized to ensure consistency across all testing sessions. The primary experimenter was responsible for operating the testing equipment, explaining the experimental tasks to participants, administering the experiment, and retrieving the experimental data. Research assistants assisted with electrode placement, helped participants wash and dry their hair after the experiment, and maintained the cleanliness of the laboratory. Participants were required to wash their hair one day before the experiment (to reduce impedance during EEG electrode placement), avoid strenuous exercise on the experiment day (to minimize interference with neural electrical activity).

Experimental stimuli were presented on a 17-inch display screen with a black background (resolution: 1024 × 768, refresh rate: 60 Hz). Emotional faces were selected from the Chinese Affective Face Picture System (CAFPS) [35], including neutral and negative faces. All images were in black and white, sized at 7 mm × 9 mm, and normalized for brightness and contrast (to control for non-emotional visual confounders). A total of 120 emotional faces were used (60 negative and 60 neutral, half male and half female), and each face was presented an equal number of times across trials (to avoid stimulus familiarity effects). Participants’ eyes were kept level with the center of the screen and positioned approximately 60 cm away from it. The E-prime software was used to present the stimuli.

The Dot-Probe Task: The experiment includes six blocks, each containing 80 trials. There are a total of 480 trials, including 360 mixed-pair trials and 60 negative-pair trials and 60 neutral-pair trials. Among the 360 mixed-pair trials, 180 trials involve the target replacing the position where a negative face was presented (congruent condition), and 180 trials involve the target replacing the position where a neutral face was presented (incongruent condition). Mixed-pair trials were the core trials for calculating attention bias, while the other two trial types served as distractor trials to reduce response anticipation. Additionally, the order of all 480 trials (including different pair types) was pseudorandomized using E-Prime 3.0’ s built-in randomization function, ensuring no sequential repetition of the same trial type or probe position exceeded three consecutive times. To further enhance design balance, each individual face (whether negative or neutral) was presented an equal number of times across the entire experiment, and the pairing of specific negative and neutral faces in mixed-pair trials was also randomized to prevent stimulus-specific effects.

The procedure for each trial is as follows: First, participants fixate on a fixation cross “+” displayed on the screen, which appears randomly for 400–600 ms. Then, the “+” disappears, and a pair of emotional faces is presented at the left and right positions of the original “+” for 400–600 ms. Subsequently, a probe stimulus (a white dot of 2 mm × 2 mm) appears at the location where one of the faces was presented, lasting for 400 ms. After the probe stimulus disappears, participants need to make a key-press judgment on the location of the probe stimulus (press F for left; J for right). After the key press, the next trial starts automatically. If no key is pressed within 1000 ms, the system will skip to the next trial automatically.

**Figure 3:**
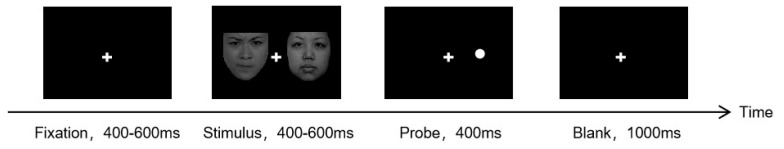
The procedure of the dot-probe task.

Before the formal experiment, participants completed 20 practice trials (with trial types identical to the formal experiment) to familiarize themselves with the task rules. The practice phase could be repeated if participants reported confusion, and the formal experiment began only after participants confirmed they understood the procedure. After the participants become familiar with the experimental procedure, they press the start button to initiate the formal experiment. The experimental procedure is shown in Figure 3.

### 2.5. Data Collection and Analysis Methods

#### 2.5.1. Behavioral Data Collection

In the dot-probe task, the behavioral data of each participant were processed as follows: (1) Trials with incorrect responses were excluded; (2) Trials with reaction times (RT) shorter than 200 ms or longer than 1000 ms were excluded; (3) RT distributions for each participant was tested using the Shapiro–Wilk test (*p* > 0.05 for all participants, indicating that RT data conformed to a normal distribution), trials with RTs beyond 3 standard deviations of the individual’s mean RT were further excluded; (4) No data transformation was applied, as the Shapiro–Wilk test confirmed the normality of RT distributions, and visual inspection of Q-Q plots further supported that RT data met the normality assumption required for subsequent parametric analyses. After the above data processing steps, the trial exclusion rate across participants ranged from 0.05% to 8.15%, with an average rate of 4.33%.

For participants included in the analysis, the dot-probe task calculated scores for attention orientation and attention disengagement. Attention orientation refers to the initial, automatic allocation of attention toward or away from negative stimuli immediately after stimulus presentation. For women with PMS during the luteal phase, excessive attention orientation toward negative stimuli manifests as a “preferential capture” of attention by negative cues, reflecting an automatic vigilance response [36]. This initial bias is considered a foundational component of PMS-related attentional dysfunction, as it determines whether attention is first drawn to negative information before conscious control is engaged. Attention disengagement describes the difficulty or ease of shifting attention away from negative stimuli once it has been captured [37]. In PMS populations, impaired attention disengagement is characterized by prolonged attention “sticking” to negative cues, even when task demands require redirecting attention to neutral or target information. This sustained fixation amplifies negative emotional experiences by prolonging exposure to aversive stimuli [13,23]. The calculation formula is as follows:

Attention Orientation = RT (neutral face pair)−RT (congruent probe);

Attention Disengagement = RT (incongruent probe)−RT (neutral face pair).

This scoring approach was adopted based on two core considerations to balance theoretical rigor and operational feasibility. First, the formula is derived from the classic component decomposition framework of attention bias [38], which conceptualizes attention bias as a combination of two sub-processes: attention orientation and attention disengagement. Although attention processing involves multiple stages, this two-component decomposition is widely used in PMS-related attention bias studies [36] for its ability to isolate the key attention sub-processes modulated by emotional states in the luteal phase. Three strategies—accuracy screening, normality testing, and outlier removal—were applied during data preprocessing to reduce noise. The low average trial exclusion rate (4.33%) verified that the formula did not excessively amplify noise, and the resulting attention orientation/attention disengagement scores remained reliable for subsequent analyses.

#### 2.5.2. EEG Data Collection

The ERP recording system of NeuroScan was used, and the EEG was recorded with a 64-channel electrode cap based on the International 10–20 system. Electrode impedance was maintained below 10 kΩ throughout the recording to ensure high-quality signal acquisition; any channels with impedance exceeding this threshold during the experiment were marked for subsequent interpolation. Matlab (R2013b), EEGLAB (v14), and ERPLAB (v7) were used for offline analysis of the continuous EEG data. With the average of the bilateral mastoids as the reference, high-pass filtering (0.1 Hz, 12 dB/octave) and low-pass filtering (30 Hz, 24 dB/octave) were carried out respectively; the filtering parameters were selected based on the frequency range of ERP components of interest (P1: 8–12 Hz; P3: 15–30 Hz), which effectively removed slow drifts and high-frequency electromyographic noise without distorting the target ERP waveforms. Taking the appearance of the emotional face pair stimuli as the marker, the continuous data was segmented within the time window from 200 ms before the appearance of the stimuli to 1000 ms after the appearance of the stimuli, and the EEG segment from −200 to 0 ms was selected as the baseline. In the dot-probe task, each participant had 480 original trials. After the above screening process, the average number of valid trials per participant was 452 ± 28 (mean ± standard deviation), with a validity rate of 94%. Independent Component Analysis (ICA) was used to remove common artifacts like electrooculographic (blinking and eye movement) artifacts, electromyographic artifacts, and electrocardiographic artifacts. Artifacts were identified and rejected based on three criteria: (1) Independent component scalp topographies matching typical artifact distributions (e.g., frontal-central maxima for eye blinks), (2) Independent component time courses correlating with visual inspection of raw EEG signals, and (3) spectral power spectra dosminated by artifact-related frequencies (e.g., >1 Hz for eye movements). Following artifact removal, channels marked with high impedance were interpolated using the spherical spline interpolation method [39], with a minimum of 8 neighboring channels used for interpolation to maintain signal integrity.

For ERP component extraction, electrode sites were selected based on previous ERP studies on attention control and emotional processing [36,40]. Specifically, electrodes Fz and Cz (frontal-central regions, reflecting central attention control processes) and PO7 and PO8 (occipital-parietal regions, corresponding to early visual sensory processing) were selected for the analysis of P1 and P3 components. The combined use of these electrodes allows for a comprehensive capture of the neurophysiological characteristics of attention bias by integrating central regulatory and peripheral perceptual processing. Latency windows for target components were defined by combining normative ERP literature and visual inspection of grand-averaged waveforms in the current study: the quantification window for P1 was set at 50–150 ms post-stimulus, and for P3 at 250–500 ms post-stimulus.

For each component, peak amplitudes were first extracted from the four electrodes (Fz, Cz, PO7, PO8) within their respective latency windows, and then the mean value of these four peak amplitudes was calculated to generate a single representative data point per component for each participant, which was used for subsequent statistical analyses. The selection of mean peak amplitude was justified by its higher sensitivity to ERP amplitude changes induced by emotional stimulus processing, while multi-electrode averaging reduces signal noise from individual channels, which is highly consistent with the core objective of this study to investigate attention bias-related neural activity changes. Finally, trials under congruent and incongruent conditions were sorted and averaged separately, and the P1 and P3 component data of the yoga intervention group and the control group were grand-averaged, respectively, to explore potential between-group differences in ERP components.

#### 2.5.3. Data Analysis Method

Descriptive statistics were used to calculate the mean and standard deviation of each variable; RM-ANOVA was used to examine the differences in variables across time, groups, and for the time × group interaction. Additionally, simple effect analysis was conducted to explore the causes of these differences; Pearson correlation analysis was used to measure the correlations between variables, and Holm–Bonferroni correction was applied to correct for multiple comparison errors. For multiple comparison corrections, the Holm–Bonferroni method was applied specifically to the pairwise comparisons derived from the significant simple effect analyses and time × group interaction effects, with the family-wise error rate (FWER) controlled at α = 0.05. The corrected *p* values for all post-hoc comparisons are reported in the table to ensure the rigor of statistical inferences.

RM-ANOVA was selected instead of a mixed-effects model because: (1) the study had a balanced design (equal sample sizes in the exercise and control groups) and no missing data, which aligns with the optimal application scenario of RM-ANOVA; (2) the primary research question focused on the fixed effects of group, time, and their interaction (rather than random effects of individual variability), and RM-ANOVA provides straightforward and interpretable estimates of these fixed effects for a 2 × 2 design; (3) the sphericity assumption was satisfied, eliminating the need for complex correction procedures often required in mixed models for unbalanced or non-spherical data.

The interpretive thresholds for effect sizes adopted in this study (specific to the fields of rehabilitation medicine, physiotherapy, and musculoskeletal sciences) are as follows: for individual difference analysis (Pearson correlation coefficient r): small effect = 0.3, medium effect = 0.5, large effect = 0.6; for between-group difference analysis (Cohen’s d or Hedges’ g): small effect = 0.1, medium effect = 0.4, large effect = 0.8 [41]; the effect size thresholds for η^2^ are: 0.01 = small effect, 0.06 = medium effect, 0.14 = large effect [42]; relevant explanations of the 95% confidence interval (95% CI) can be found in the cited literature [43].

## 3. Results

All behavioral data (attention orientation, attention disengagement) and EEG data (P1, P3 components) were analyzed using the RM-ANOVA model. Results of mauchly’s test of sphericityconfirmed that the sphericity assumption was satisfied for all variables (attention orientation: χ^2^ = 0.82, df = 1, *p* = 0.37 > 0.05; attention disengagement: χ^2^ = 0.91, df = 1, *p* = 0.34 > 0.05; P1 component: χ^2^ = 0.78, df = 1, *p* = 0.38 > 0.05; P3 component: χ^2^ = 0.85, df = 1, *p* = 0.36 > 0.05). Thus, no Greenhouse-Geisser or Huynh-Feldt correction was required. Partial eta squared (η_p_^2^) with 95% confidence intervals was reported for all main effects and interaction effects to quantify the magnitude of effects. Simple effect analyses were performed, with the Holm–Bonferroni correction applied to control the family-wise error rate (FWER = 0.05), and the corrected *p*-values (pcorr) were reported for all pairwise comparisons derived from the simple effects. Specifically, the correction targeted two types of post-hoc comparisons: (1) When the group factor was fixed to examine within-group differences across time points: Corrected *p*-values were reported for comparisons of pre-intervention vs. post-intervention scores within the yoga group (for attention orientation, attention disengagement, P1 amplitude, and P3 amplitude) and within the control group (for the same four variables). (2) When the time factor was fixed to examine between-group differences at each time point: Corrected *p*-values were reported for comparisons of yoga group vs. control group scores at the pre-intervention time point (for the four variables) and at the post-intervention time point (for the same four variables).

### 3.1. Behavioral Data of Attention Bias

The RM-ANOVA of attention orientation showed that the main effect of time was significant, F(1, 62) = 14.40, *p* < 0.001, η_p_^2^ = 0.19; the main effect of group was not significant, F(1, 62) = 2.16, *p* = 0.15, η_p_^2^ = 0.03; the time × group interaction was significant, F(1, 62) = 61.14, *p* < 0.001, η_p_^2^ = 0.50. The RM-ANOVA of attention disengagement showed that the main effect of time was significant, F(1, 62) = 20.24, *p* < 0.001, η_p_^2^ = 0.25; the main effect of group was significant, F(1, 62) = 5.94, *p* = 0.02, η_p_^2^ = 0.08; the time × group interaction was significant, F(1, 62) = 42.44, *p* < 0.001, η_p_^2^ = 0.41.

**Figure 4:**
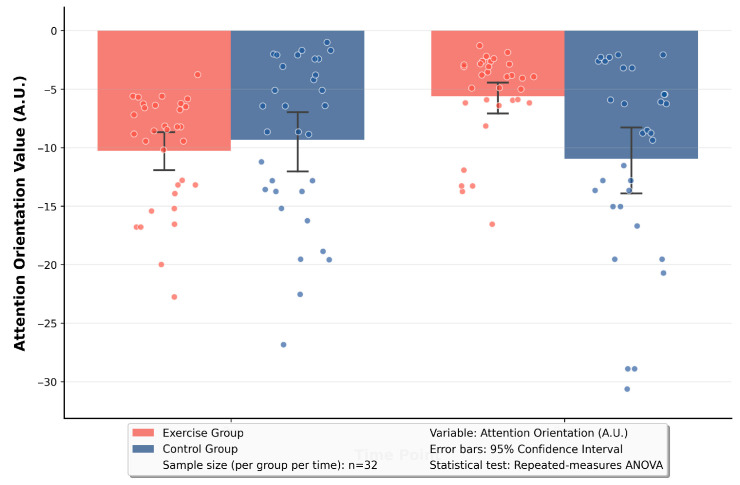
Pre-test vs. post-test comparison of attention orientation in two groups.

**Figure 5:**
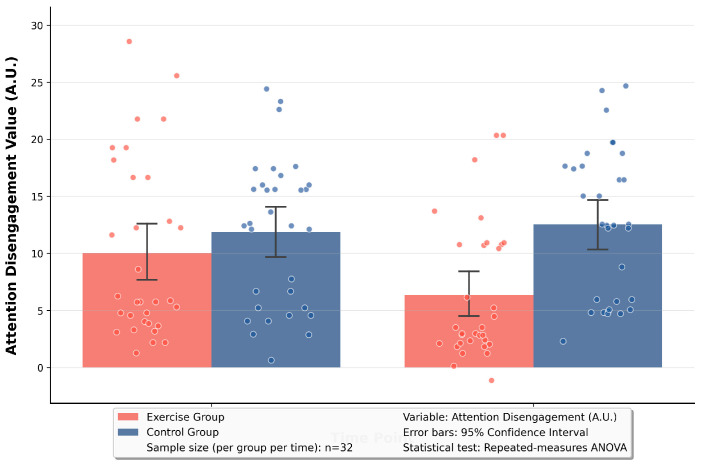
Pre-test vs. post-test comparison of attention disengagement in two groups.

Based on the significant time × group interaction effect revealed by the RM-ANOVA, simple effect analyses were conducted on attention orientation and attention disengagement. The results are as follows: In terms of the simple effect of time, for attention orientation: the exercise group improved from a significant negative bias (−10.27) at the baseline to a reduced negative bias (−5.60) at the 12th week, with t = −7.33, *p* < 0.001. This indicated that the exercise group showed a significant decrease in attention orientation toward negative stimuli. For attention disengagement: the exercise group changed from a state of difficulty in disengagement (15.32) at the baseline to enhanced disengagement ability (8.76) at the 12th week, with t = 6.89, *p* < 0.001. This suggested that the exercise group had a significant improvement in the efficiency of disengaging attention from negative stimuli. In terms of the simple effect of group, for attention orientation: at the 12th week, the negative attention bias of the exercise group (−5.60) was significantly weaker than that of the control group (−7.98), with t = 2.58, *p* = 0.04. This demonstrated that the exercise group had a significantly lower attentional preference for negative stimuli compared with the control group. For attention disengagement: at the 12th week, the difficulty of attention disengagement in the exercise group (8.76) was significantly lower than that in the control group (13.92), with t = −5.27, *p* < 0.001. This indicated that the exercise group had a significantly stronger ability to disengage attention from negative stimuli than the control group. See Table 2 and Figure 4 and Figure 5 for details.

### 3.2. EEG Data of Attention Bias

The RM-ANOVA of P1 showed that the main effect of time was significant, F(1, 62) = 24.51, *p* < 0.001, η_p_^2^ = 0.28; the main effect of group was significant, F(1, 62) = 5.51, *p* = 0.02, η_p_^2^ = 0.08; and the time × group interaction was significant, F(1, 62) = 32.14, *p* < 0.001, η_p_^2^ = 0.34. The analysis of P3 showed that the main effect of time was significant, F(1, 62) = 7.32, *p* = 0.009, η_p_^2^ = 0.11; the main effect of group was significant, F(1, 62) = 10.40, *p* = 0.002, η_p_^2^ = 0.14; and the time × group interaction was significant, F(1, 62) = 11.80, *p* = 0.001, η_p_^2^ = 0.16.

**Figure 6:**
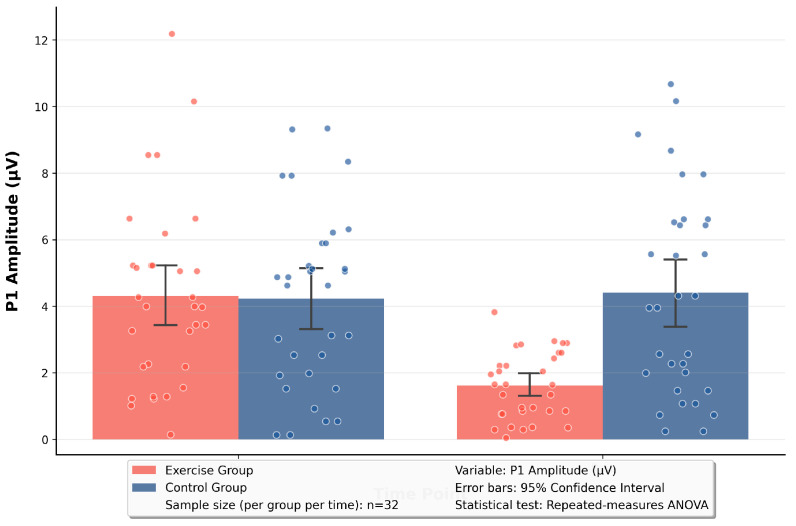
Pre-test vs. post-test comparison of P1 in two groups.

**Figure 7:**
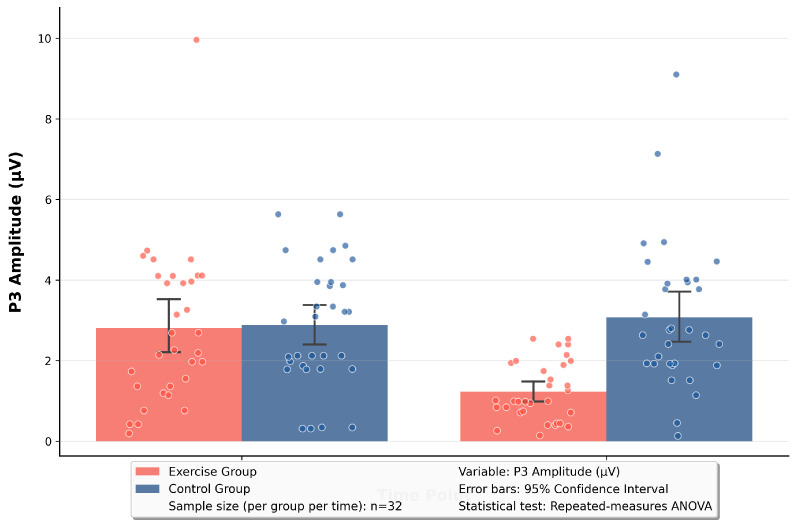
Pre-test vs. post-test comparison of P3 in two groups.

Based on the significant time × group interaction effect revealed by the RM-ANOVA, simple effect analyses were performed on the P1 and P3 components. The results are as follows: In terms of the simple main effect of time, for the P1 component, the exercise group exhibited a reduction in amplitude from excessive activation of early sensory attention (5.82 μV) at baseline to activation normalization (3.21 μV) at the 12th week, with t = 8.94, *p* < 0.001. This indicated that the exercise group showed a significant decrease in early sensory vigilance toward negative stimuli. For the P3 component: the exercise group’s amplitude declined from abnormally enhanced late cognitive attention (2.80 μV) at baseline to cognitive regulation normalization (1.23 μV) at the 12th week, with t = 4.42, *p* = 0.002. This suggested that the exercise group had a significant optimization in late cognitive evaluation and resource allocation for negative stimuli. In terms of the simple main effect of group, for the P1 component: at the 12th week, the early sensory activation of the exercise group (3.21 μV) was significantly lower than that of the control group (5.39 μV), with t = −7.62, *p* < 0.001. This demonstrated that the exercise group’s early sensory vigilance toward negative stimuli was significantly weaker than that of the control group. For the P3 component: at the 12th week, the late cognitive activation of the exercise group (1.23 μV) was significantly lower than that of the control group (2.51 μV), with t = −4.15, *p* < 0.001. This indicated that the exercise group allocated significantly fewer late cognitive resources to negative stimuli compared with the control group. See Table 3 and Figure 6 and Figure 7 for details.

### 3.3. Brain Waves and Topographic Maps Associated with Attention Bias

**Figure 8:**
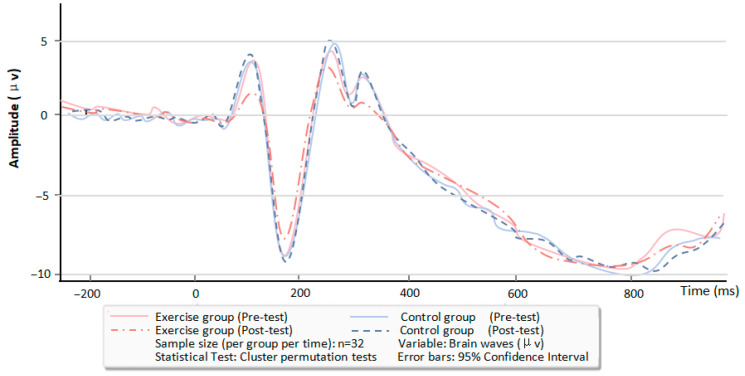
Pre-test vs. post-test comparison of brain waves in two groups.

**Figure 9:**
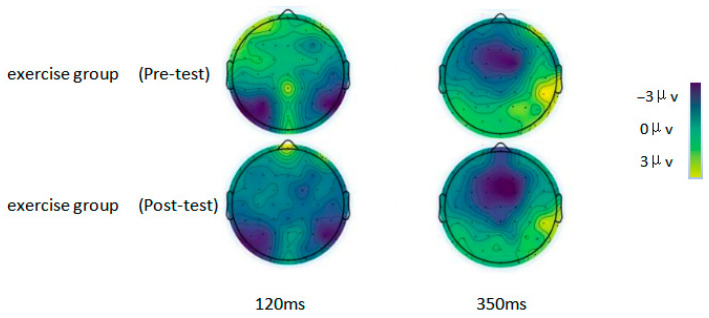
Pre-test vs. post-test comparison of topographic maps (120 ms and 350 ms) in the exercise group.

To statistically evaluate EEG differences between the two groups (with comparisons oriented toward the exercise group versus the control group) while mitigating Type I errors associated with multiple comparisons, a cluster-based permutation test was implemented. The analytical procedure was structured as follows: first, independent-samples *t*-tests were performed to compare amplitude data between the exercise and control groups at each individual time point. Consecutive time points yielding significant group differences were then aggregated into “clusters,” and the sum of the absolute t-values across all time points within each cluster was computed as the cluster-level test statistic. Next, a null distribution was generated via 1000 iterations of data permutation, and the 95th percentile of this null distribution was set as the critical threshold (α = 0.05). Clusters in the original data that exceeded this critical threshold were classified as statistically significant. Permutation test results revealed that after the 12-week intervention, the exercise group had a significantly lower P1 amplitude compared to the control group (cluster statistic = 28.7, permutation test *p* = 0.02). Similarly, the P3 amplitude in the exercise group was significantly reduced relative to the control group following the intervention (cluster statistic = 25.6, permutation test *p* = 0.03). In the dot-probe task, the average peak amplitudes of the P1 and P3 components were observed at approximately 120 ms and 350 ms, respectively. Following the 12-week intervention, the exercise group exhibited a significant reduction in the average peak amplitudes of both P1 and P3 during the dot-probe task. Consistent with this finding, brain topographic maps demonstrated decreased activation levels at the 120 ms (P1-related) and 350 ms (P3-related) time points in the exercise group relative to its baseline (see Table 4 and Figure 8 and Figure 9 for detailed results).

### 3.4. Corresponding Analysis

Pearson correlation analysis was used to examine the correlation between the behavioral data and the EEG data of attention bias. The results showed that: attention orientation was significantly negatively correlated with P1 (rs = −0.500, *p* < 0.001), with a 95% CI [−0.668, −0.283]; attention disengagement was significantly positively correlated with P3 (rs = 0.251, *p* = 0.046), with a 95% CI [−0.002, 0.473].

In addition, within the behavioral data of the dot-probe task, attention orientation was significantly negatively correlated with attention disengagement (rs = −0.596, *p* < 0.001, 95% CI [−0.738, −0.404]); within the EEG data of the dot-probe task, P1 was significantly positively correlated with P3 (rs = 0.360, *p* = 0.003, 95% CI [0.122, 0.555]).

## 4. Discussion

To investigate the regulatory effect of yoga on attentional dysfunction in women with PMS during the luteal phase, this study implemented a 12-week yoga intervention (twice weekly, 60 min per session, integrating asana practice, breath regulation, and mindfulness meditation). Combined with the dot-probe task (for assessing behavioral attention bias) and EEG recording (for measuring neural activity), the study systematically analyzed the regulatory impacts of yoga on core attentional dimensions (attention orientation and attention disengagement) and electrophysiological indices (the P1 component, an early marker of sensory attention, and the P3 component, a late marker of cognitive attention). The results confirmed that yoga exerts dual regulatory effects on abnormal attentional function at both the behavioral and neural levels.

In terms of behavioral regulatory effects, there was no significant change in attention orientation in the control group after the intervention (*p* > 0.05), whereas the yoga group exhibited a marked reduction in excessive attention orientation toward negative stimuli: the mean score of attention orientation increased from −10.27 ± 4.78 to −5.60 ± 3.93 (t = −7.327, *p* < 0.001). This finding indicates that yoga can effectively attenuate the automatic capture of negative information in women with PMS, reduce the over-allocation of attentional resources to negative stimuli, and break the characteristic pattern of “excessive prioritization of negative cues” in attention orientation during the luteal phase. This result is consistent with the research conclusion by Jha et al. [44,45] that mindfulness-based interventions can improve emotional attention bias; their study also confirmed that intervention approaches integrating breath regulation and attention training can significantly reduce individuals’ attentional preference for negative stimuli. Regarding attention disengagement, no significant changes were observed in the control group (*p* > 0.05), while the yoga group demonstrated significantly improved flexibility in shifting attention from negative to neutral stimuli, with the mean score of attention disengagement decreasing from 10.02 ± 7.74 to 6.36 ± 5.97 (t = 6.252, *p* < 0.001). This improvement may be attributed to the “attentional control training” inherent in yoga practice: for example, balance asanas require practitioners to sustain focus on bodily sensations and breathing rhythms; when the mind wanders, individuals must actively redirect their attention back to the task. This repeated “volitional attentional shifting” can gradually strengthen the capacity for attention disengagement [40,41], ultimately breaking the vicious cycle of “being trapped by negative stimuli and unable to disengage” experienced by women with PMS during the luteal phase. Notably, the ameliorative effect of yoga on attention disengagement in this study is consistent with the research trend reported by Epp et al. that “emotion regulation interventions can enhance attentional flexibility in PMS populations” [46]. However, this study further clarifies the specificity of this effect during the luteal phase and its association with yoga intervention.

At the neural regulatory mechanism level, the yoga group displayed a significant reduction in P1 amplitude in response to negative stimuli after the intervention, decreasing from 4.31 ± 2.79 μV to 1.61 ± 1.01 μV (t = 5.709, *p* < 0.001), while no such change was detected in the control group (*p* > 0.05). The reduced P1 amplitude in the yoga group suggests that yoga inhibits the over-allocation of early sensory attention to negative stimuli, thereby weakening excessive automatic orientation toward negative cues at the neural level. A potential mechanism underlying this effect may be linked to yoga-based breath regulation, which can stimulate the vagus nerve and reduce the excitability of the sympathetic nervous system [47,48,49]. This, in turn, suppresses the hyperactivation of the visual cortex and parietal cortex in women with PMS when exposed to negative cues, mitigating the “biased allocation” of early attentional resources to negative information. This neural regulatory pattern complements the finding by Baker et al. that “neural activity related to negative stimulus processing is enhanced in women with PMS during the luteal phase,” confirming that yoga can reverse this abnormal neural response [50]. Consistent with the changes in P1 amplitude, the yoga group also exhibited a significant decrease in P3 amplitude in response to negative stimuli post-intervention, falling from 2.80 ± 1.93 μV to 1.23 ± 0.73 μV (t = 4.424, *p* < 0.001), whereas the control group showed no significant alterations (*p* > 0.05). The reduced P3 amplitude in the yoga group reveals the ameliorative effect of yoga on late-stage cognitive regulation of attention. The underlying mechanism may stem from mindfulness meditation during the intervention, which enhances the functional coordination between the brain’s cognitive control network and attentional regulation network [51,52]. This coordination improves the ability of women with PMS to inhibit interference from negative stimuli and actively shift attention to neutral cues. This neural regulatory pattern of “early attenuation + late modulation” corresponds to the two sequential stages of negative attention bias expression (automatic capture → sustained attention), indicating that yoga can simultaneously modulate both the early sensory and late cognitive stages of attentional processing. This finding is highly consistent with the viewpoint proposed by Craner et al. that “emotion-related attentional abnormalities require intervention from the perspective of multi-stage processing” [53,54].

In terms of statistical effect size, RM-ANOVA results revealed significant time × group interaction effects for attention orientation, attention disengagement, P1 amplitude, and P3 amplitude, with all partial eta-squared values (η_p_^2^) exceeding the threshold for large effect sizes (0.14): 0.50 for attention orientation, 0.41 for attention disengagement, 0.34 for P1 amplitude, and 0.16 for P3 amplitude. Notably, the η_p_^2^ values for the interaction effects of attention orientation, attention disengagement, and P1 amplitude (ranging from 0.34 to 0.50) were significantly higher than that for P3 amplitude (0.16). These results suggest that the synergistic effect of yoga intervention and time plays a more prominent role in regulating attention orientation, attention disengagement, and early sensory attention, whereas its modulation of late cognitive attention is relatively moderate. Nevertheless, all effects reached the large effect size level, further verifying the reliability and specificity of the intervention outcomes. Regarding correlation verification, correlation analysis confirmed the robustness of the “behavior-neural” regulatory loop: attention orientation was significantly negatively correlated with P1 amplitude (r_s_ = −0.500, *p* < 0.001, 95% CI [−0.668, −0.283]), indicating that greater reductions in P1 amplitude correspond to more pronounced improvements in attention orientation. Additionally, attention disengagement was significantly positively correlated with P3 amplitude (r_s_ = 0.251, *p* = 0.046, 95% CI [−0.002, 0.473]), suggesting that larger decreases in P3 amplitude are associated with more significant enhancements in attention disengagement. These correlations validate the close link between yoga-induced neural changes and behavioral improvements, clarifying that yoga ameliorates negative attention bias by coordinately regulating early sensory and late cognitive attentional processes. This provides empirical support for the theoretical construction of the “exercise-brain-attention” regulatory pathway. It also fills the research gap in the existing field of PMS cognitive intervention regarding “insufficient evidence for neural-behavioral associations” [55].

In conclusion, this study not only confirms the dual regulatory effect of yoga intervention on attentional dysfunction in women with PMS during the luteal phase at both the behavioral and neural levels but also reveals its synergistic regulatory mechanism of “early sensory inhibition—late cognitive modulation.” Yoga not only modulates the abnormal patterns of PMS-related attention bias (excessive orientation toward and difficulty disengaging from negative stimuli) but also blocks the vicious cycle of “excessive attention bias—negative emotion” by reducing hypervigilance toward negative stimuli. It echoes the existing research system on improving emotional cognitive function through exercise interventions [7] and further clarifies the intervention value of yoga in specific populations with PMS and specific physiological stages.

## 5. Research Limitations and Future Prospects

First, there are limitations in participant recruitment. The sample size of this study was restricted to a homogeneous group (female college students), which may not fully represent the broader population of women with PMS (e.g., those of different ages, occupational backgrounds, or PMS symptom severities); this homogeneity could compromise the external validity and generalizability of the current findings. To address this limitation, future research should expand to larger-scale samples and include more demographically diverse populations (e.g., working women, middle-aged women with PMS) to verify the robustness and general applicability of the observed effects of yoga on attention bias.

Second, in terms of research methodology, this study was unable to rule out the potential impact of the placebo effect, which in turn may have influenced attention-related behavioral and neurological indicators. Moreover, the causal inference regarding the regulatory effect of yoga on attentional dysfunction may have overlooked potential confounding factors. For instance, the expectation effect and the level of attention provided by instructors could both be independent factors affecting the intervention outcomes. Although the dot-probe task employed in this study has a notable advantage in temporal precision, it cannot directly localize neural activity in specific brain regions. Future research should integrate the dot-probe task with neuroimaging technologies such as functional magnetic resonance imaging (fMRI), thereby enabling a more comprehensive exploration of how yoga modulates neural activity patterns and brain structural plasticity in women with PMS. Future research will also consider incorporating questionnaires or experimental tasks related to cognitive reappraisal to further elucidate the mechanistic role of yoga in preventing negative emotional experiences associated with PMS.

Third, integrated analysis of hormonal data, questionnaire data, and experimental data was not performed. In this study, participants’ hormone levels (estrogen and progesterone) were measured at pre-test and post-test, and PSST questionnaire data were collected. Future research directions should involve incorporating these data for more in-depth analyses, such as mediating/moderating effect analyses of hormone levels and experimental data, and correlation analyses between the 19 items (based on the PSST scale) and experimental data. Some women with PMS may exhibit deviations in hormone levels during the luteal phase, and it cannot be ruled out that yoga intervention regulates attentional function by restoring normal hormone levels in participants during the luteal phase.

## 6. Conclusions

The present study confirms that regular yoga exerts a regulatory effect on the attention bias of women with PMS, and yoga ameliorates the excessive attentional focus on negative stimuli that is characteristic of this population during the luteal phase. These findings provide new insights into the PMS-related attentional abnormalities and corresponding intervention strategies, while also offering scientific evidence for the application of yoga in the intervention of emotion-related cognitive disorders in women.

## Figures and Tables

**Table 1:** Baseline characteristics of participants.

	Total (n = 64)	Exercise Group (n = 32)	Control Group (n = 32)	t	*p*
Age (years)	19.14 ± 0.35	19.16 ± 0.37	19.13 ± 0.34	0.35	0.72
Height (cm)	164.31 ± 4.78	164.59 ± 5.30	164.03 ± 4.26	0.47	0.64
Weight (kg)	54.52 ± 6.10	54.72 ± 6.04	54.31 ± 6.24	0.27	0.79
BMI (kg/m^2^)	20.19 ± 2.11	20.20 ± 2.01	20.19 ± 2.24	0.01	0.99
Psychological Symptoms	13.94 ± 2.40	14.28 ± 3.09	13.59 ± 1.39	1.15	0.26
Physical Symptoms	9.83 ± 2.12	10.09 ± 2.01	9.56 ± 2.23	1.00	0.32
Symptomatic Impact	6.89 ± 2.81	6.63 ± 2.84	7.16 ± 2.80	−0.76	0.45
All Symptoms and Impacts	30.70 ± 4.47	31.09 ± 4.99	30.31 ± 3.91	0.70	0.48
Exercise Intensity	2.05 ± 0.58	2.09 ± 0.59	2.00 ± 0.57	0.65	0.52
Exercise Time	3.90 ± 0.85	3.97 ± 0.86	3.84 ± 0.85	0.59	0.56
Exercise Frequency	3.89 ± 1.39	3.94 ± 1.37	3.84 ± 1.44	0.27	0.79

**Table 2:** Attention orientation and attention disengagement: group and time-related comparisons.

Variable	Specific Comparison	Key Values	t	Corrected p	95% CI
Attention Orientation	Exercise group (pre → post)	Pre: −10.27 ± 4.78 Post: −5.60 ± 3.93	−7.33	<0.001	[−6.12, −2.92]
	Control group (pre → post)	Pre: −8.15 ± 4.22 Post: −7.98 ± 4.05	−0.35	0.73	[−1.89, 1.55]
	Pre-test (exercise vs. control)	Exercise: −10.27 ± 4.78 Control: −8.15 ± 4.22	−2.07	0.12	[−4.45, 0.21]
	Post-test (exercise vs. control)	Exercise: −5.60 ± 3.93 Control: −7.98 ± 4.05	2.58	0.04	[0.45, 4.31]
Attention Disengagement	Exercise group (pre → post)	Pre: 15.32 ± 5.11 Post: 8.76 ± 3.89	6.89	<0.001	[4.32, 8.70]
	Control group (pre → post)	Pre: 14.85 ± 4.87 Post: 13.92 ± 4.53	1.21	0.52	[−0.78, 2.64]
	Pre-test (exercise vs. control)	Exercise: 15.32 ± 5.11 Control: 14.85 ± 4.87	0.38	0.94	[−1.92, 2.86]
	Post-test (exercise vs. control)	Exercise: 8.76 ± 3.89 Control: 13.92 ± 4.53	−5.27	<0.001	[−7.21, −3.01]

**Table 3:** P1 and P3: group and time-related comparisons.

Variable	Specific Comparison	Key Values	t	Corrected *p*	95%CI
P1	Exercise group (pre → post)	Pre: 5.82 ± 1.63 Post: 3.21 ± 1.05	8.94	<0.001	[1.98, 3.24]
	Control group (pre → post)	Pre: 5.67 ± 1.58 Post: 5.39 ± 1.42	1.03	0.61	[−0.35, 0.91]
	Pre-test (exercise vs control)	Exercise: 5.82 ± 1.63 Control: 5.67 ± 1.58	0.41	0.94	[−0.65, 0.95]
	Post-test (exercise vs control)	Exercise: 3.21 ± 1.05 Control: 5.39 ± 1.42	−7.62	<0.001	[−2.85, −1.51]
P3	Exercise group (pre → post)	Pre: 2.80 ± 1.93 Post: 1.23 ± 0.73	4.42	0.002	[0.81, 2.33]
	Control group (pre → post)	Pre: 2.67 ± 1.85 Post: 2.51 ± 1.72	0.57	0.85	[−0.62, 0.94]
	Pre-test (exercise vs control)	Exercise: 2.80 ± 1.93 Control: 2.67 ± 1.85	0.28	0.97	[−0.85, 1.11]
	Post-test (exercise vs control)	Exercise: 1.23 ± 0.73 Control: 2.51 ± 1.72	−4.15	<0.001	[−1.85, −0.61]

**Table 4:** Statistical results of cluster permutation tests for P1/P3 EEG Components.

Variable	Analysis Time Point	Time Window (ms)	Number of Permutation Tests	Cluster Statistic (Sum of t)	*p*
P1	Pre-test	100–150	1000	12.8	0.22
	Post-test	100–150	1000	28.7	0.02
P3	Pre-test	300–500	1000	14.5	0.13
	Post-test	300–500	1000	25.6	0.03

## Data Availability

The datasets generated and analyzed during this study, including de-identified baseline characteristics, preprocessed EEG data, and statistical outputs. Data sharing complies with institutional ethics guidelines. The data that support the findings of the study are available from the corresponding author upon reasonable request.

## Abbreviations

The following abbreviations are used in this manuscript:

PMS	Premenstrual Syndrome
RM-ANOVA	Repeated-Measures Analysis of Variance
EEG	Electroencephalogram
ERP	Event-related Potentials
PSST	Premenstrual Symptoms Screening Tool
PARS	Physical Activity Rating Scale
PMDD	Premenstrual Dysphoric Disorder
BMI	Body Mass Index
CAFPS	Chinese Affective Face Picture System
RT	Reaction Times
ICA	Independent Component Analysis
fMRI	functional Magnetic Resonance Imaging
